# Direct observation of cytoskeleton-dependent trafficking of miRNA visualized by the introduction of pre-miRNA

**DOI:** 10.1016/j.isci.2024.108811

**Published:** 2024-01-05

**Authors:** Toshinari Ishikawa, Ko Sugawara, Junwei Zhang, Takashi Funatsu, Kohki Okabe

**Affiliations:** 1 Graduate School of Pharmaceutical Sciences, The University of Tokyo, 7-3-1 Hongo, Bunkyo-ku, Tokyo 113-0033, Japan; 2 JST, PRESTO, 4-8-1 Honcho, Kawaguchi, Saitama 332-0012, Japan

**Keywords:** Nucleic acids, Molecular physiology, Molecular biology, Biomechanics

## Abstract

MicroRNA (miRNA) plays physiologically and pathologically important roles in post-transcriptional regulation. Although miRNA has been suggested to dynamically interact with cellular organelles, the dynamicity of intracellular miRNA behavior has remained unclear. Here, by introducing fluorescently labeled pre-miRNA into living cells, we improved the miRNA visualization method using exogenous miRNA precursors. Through the combination of our miRNA visualization method and single-molecule sensitive fluorescence microscopy, we quantitatively analyzed the process of miRNA maturation. Furthermore, single-particle tracking of fluorescent miRNA in cells revealed the directed movements of miRNA on cytoskeletal components (i.e., microtubules and actin filaments). Our results also suggest that cytoskeleton-dependent miRNA trafficking is associated with the interaction of miRNAs with the nucleus and the endoplasmic reticulum/Golgi apparatus. Our method should facilitate the elucidation of the mechanism and physiological significance of the subcellular localization and organelle interaction of miRNA.

## Highlights

• Introduction of fluorescent pre-miRNA into living cells efficiently visualized miRNAs
• Intracellular maturation processes of pre-miRNA were tracked in real time
• Single particle tracking showed miRNA movement in association with the cytoskeletons
• Cytoskeletons may contribute to intracellular trafficking of miRNAs

## Graphical abstract

**Figure:**
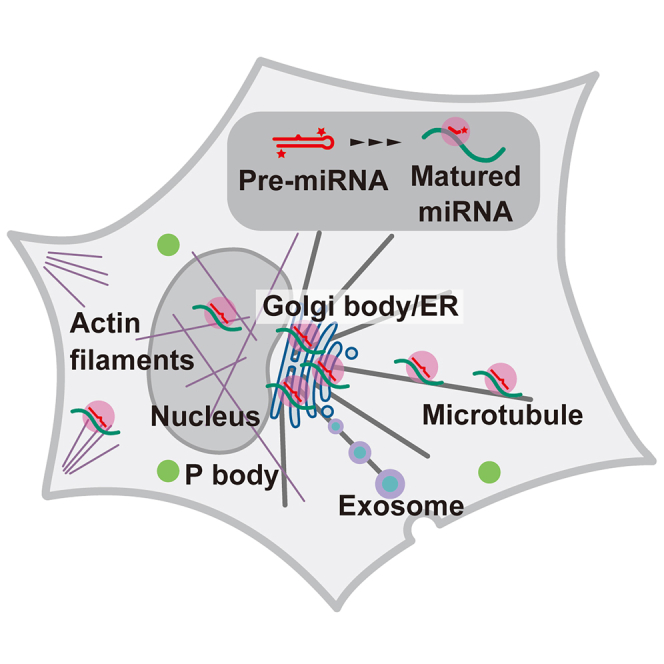

## Introduction

MicroRNAs (miRNAs) are evolutionarily conserved small non-coding RNAs that repress the translation of specific mRNAs of various genes in eukaryotes, and their dysfunction has been suggested to cause various diseases, including cancer, neurodegenerative diseases, and cardiovascular diseases.^1^^,^^2^^,^^3^^,^^4^^,^^5^ In general, miRNA guide strands bind to partially complementary sequences in the 3′-untranslated region of mRNAs, causing translational repression, cleavage, or deadenylation of the target mRNA.^6^^,^^7^ Recently, it has emerged that miRNA functions are closely related to intracellular organelles and intracellular processes.^8^^,^^9^ In addition to mRNA cleavage at the processing body (PB),^10^^,^^11^ diverse interactions of intracellular miRNAs with organelles have been studied. Aside from post-transcriptional gene silencing (PTGS) in the cytoplasm, miRNAs have been found in major cellular compartments such as the endoplasmic reticulum (ER) membrane, nucleus, nucleoli, and mitochondria.^12^ For example, miRNAs function in the nucleus, including in transcription, alternative splicing, and DNA repair,^12^^,^^13^^,^^14^ and to regulate mitochondrial translation by being transported from the cytoplasm to mitochondria.^15^ Furthermore, miRNAs have been implicated in various functions, including sequence-dependent sorting into exosomes,^16^^,^^17^ cooperating with the Golgi apparatus,^18^ and participating in ER stress and unfolded protein response.^19^ These dynamic interactions of miRNA with various organelles indicate the coordinated distribution and trafficking of miRNA relevant to its state and function in cells.

Despite these suggested spatiotemporal dynamic interactions of miRNAs in cells, the direct association of miRNA with organelles and the mechanism that determines how miRNA is distributed in cells remain unclear. The lack of understanding of miRNA behavior in cells is due to the fact that conventional methods to visualize intracellular miRNAs require cell fixation or alternatively involve observation of miRNA probe dynamics^20^; cell fixation does not allow analysis of miRNA dynamics, and probes that dissociate from miRNAs after reaction with miRNAs do not provide spatial information about miRNAs, thereby providing limited insight into intracellular miRNA localization and trafficking. This barrier is likely to delay our understanding of the molecular mechanisms of intracellular miRNAs.^21^

The visualization and direct observation of miRNA in cells are promising tools to understand its behavior and interaction with cellular components.^8^ Recently, several reports have demonstrated that introducing a synthesized fluorescent miRNA/miRNA∗ duplex (miRNA duplex) into living cells led to its incorporation into endogenous RNA-induced silencing complex (RISC) and to its demonstration of the ability to repress translation, showing the feasibility of this strategy to visualize miRNAs in single living cells and thereby providing new biological insights.^11^^,^^22^^,^^23^^,^^24^^,^^25^

In this study, we chose miRNAs known for their conservation among species and clinical importance (e.g., let-7a-1miRNA) as targets for observation. Because the introduction of unlabeled pre-miRNA exhibits higher translational repression ability than miRNA duplex,^26^^,^^27^ we developed a more effective method for visualizing miRNAs by introducing fluorescently labeled pre-miRNAs directly into living cells. Furthermore, quantitative observations using single-molecule detection techniques such as fluorescence cross-correlation spectroscopy (FCCS) suggest that the introduced pre-miRNA undergoes maturation through an endogenous biogenesis pathway (i.e., duplex dissociation, mRNA binding, and intracellular delivery) in living cells. Finally, direct observation of miRNA by fluorescence single-particle tracking (SPT) revealed that miRNA moved along cytoskeletal components including actin filaments and microtubules, suggesting that this movement contributes to the intracellular delivery of miRNA from the perinuclear region where with densely packed endoplasmic reticulum and Golgi apparatus.

## Results

### Comparison of stability of fluorescently labeled miRNA precursors

**Figure 1:**
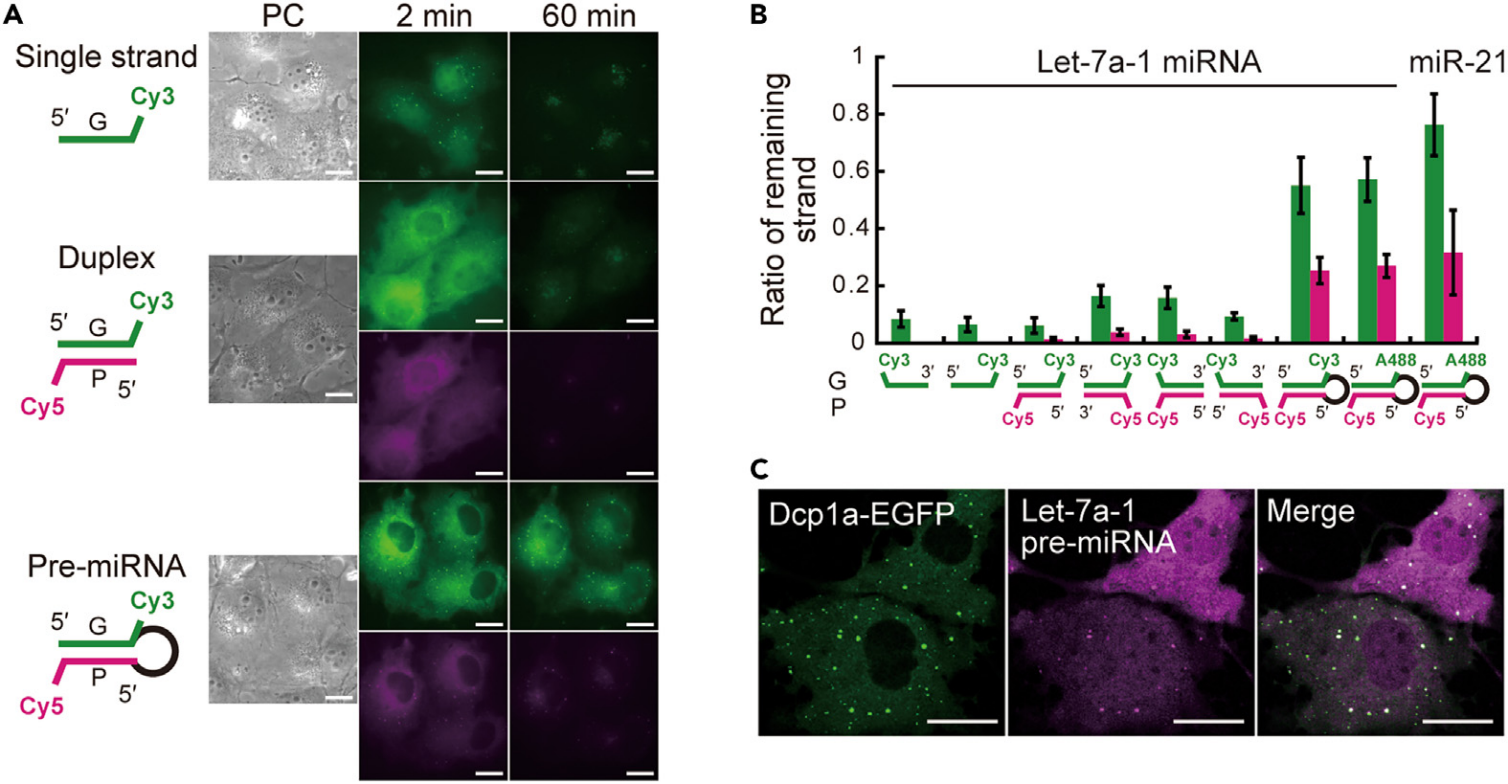
Comparison of remaining efficiency in living cells upon introduction of various fluorescently labeled miRNA precursors (A) Phase contrast (PC) images and epi-fluorescent images of the Cy3-and/or Cy5-labeled synthetic let-7a-1 miRNA precursors [single guide strand (top), duplex (middle), pre-miRNA (bottom)] microinjected into the cytoplasm. The guide strands were labeled with Cy3 and the passenger strands with Cy5. (B) The proportion of remaining strands in the cytoplasm. The results are shown for nine miRNA precursors with guide strands labeled with Cy3 or Alexa 488 and/or passenger strands labeled with Cy5. The proportion of total fluorescence intensity in the cytoplasm at 60 min after microinjection to that at 2 min after microinjection is shown. Guide (green) and passenger (magenta) strands are shown. Error bars represent SD (n = 10–14 cells). (C) Colocalization of P-body and introduced pre-miRNA. Confocal fluorescence images of P-body visualized with Dcp1a-EGFP (left), let-7a-1 pre-miRNA (Cy5-labeled guide strand, middle), and their merged image (right). The cell-to-cell variation in fluorescence intensity is due to the difference in the amount of miRNA introduced by microinjection. G and P stand for guide and passenger sequences of miRNA, respectively. Scale bars, 20 μm.

There are several forms of miRNA precursors in the cytoplasm, which gradually mature through multiple processing steps: pre-miRNA, miRNA duplex, and single-stranded miRNA. To evaluate suitable miRNA precursors for visualization, we tested their stability in cells. Introduced miRNA precursors were assumed to be depleted unless they formed stable complexes with proteins like RNA-induced silencing complex (RISC). After microinjecting the three kinds of fluorescently labeled miRNA precursors (pre-miRNA, miRNA duplex, and single-stranded miRNA, Table S1) into living cells, we acquired fluorescent images of each one at various time points. Representative results for each let-7a-1 miRNA precursor are shown in Figure 1A. The amount of introduced miRNA was determined according to the previous work.^24^ The stability of the miRNA precursors was compared by calculating the ratio of fluorescence signal remaining in the cytoplasm (Figure 1B). The signal of single-stranded miRNA was greatly reduced immediately after introduction, most of the fluorescence was depleted 60 min after microinjection, indicating that little of the introduced single-stranded miRNA was processed into a stable complex through the endogenous biogenesis pathway. Compared to single-stranded miRNAs, miRNA duplexes exhibited higher intracellular retention both immediately after introduction and after 60 min. In particular, the guide strand was more stable than the passenger strand, suggesting that a portion of the guide strand of the duplex was incorporated into a stable complex. This is consistent with the findings of Pitchiaya et al.,^24^^,^^25^ who also observed that the miRNA duplex had better intracellular stability than the single-stranded miRNA. It should also be noted that the difference in the ratio of each duplex indicates that the combination of fluorescent labeling sites (especially that one side of the duplex is unlabeled) is important for stable complex formation. Meanwhile, the introduced pre-miRNA showed even higher retention at 60 min after microinjection, and its guide strand was stable in the cytoplasm. This difference in the intracellular retention of introduced miRNA precursors was also observed in HeLa cells, a human cell line (Figure S1). Furthermore, longer-time observation of fluorescently labeled pre-miRNA showed that the amount of the pre-miRNA became stable from 2 h to at least 12 h after microinjection (Figure S2), consistent with the previous finding that intracellular miRNA had a long half-life.^28^ Because exogenous naked pre-miRNAs are easily degraded, it is expected that some of the introduced exogenous pre-miRNAs will be destroyed before being processed into stable complexes via the endogenous biosynthetic pathway, whereas molecules that have interacted with endogenous factors are expected to remain stable, as shown in Figure S2.

Next, we investigated the signal of let-7a-1 miRNA observed in the nucleus. The results showed that both introduced single-stranded miRNA and miRNA duplex accumulated in the nucleus immediately after microinjection, in accordance with previous work showing that single-stranded RNAs nonspecifically accumulated in the nucleus.^29^ In contrast, a small amount of introduced pre-miRNA accumulated in the nucleus immediately after introduction (Figure S3). This suggests that, immediately after introduction, pre-miRNA accumulates less in the nucleus and more in the cytoplasm. The introduced pre-miRNA was observed as bright spots scattered in the cytoplasm at both 2 and 60 min after introduction, and they were identified as PB by colocalization with the PB marker, EGFP-Dcp-1a (Figure 1C), which is consistent with previous findings.^10^^,^^11^

Taken together, the above results demonstrate the high retention of introduced pre-miRNAs in the cytoplasm for long periods of time, which enables various intracellular analyses of miRNA, including single-molecule sensitive imaging methods that require the accumulation of a large number of photons.

### Confirmation of the maturation of introduced miRNA

**Figure 2:**
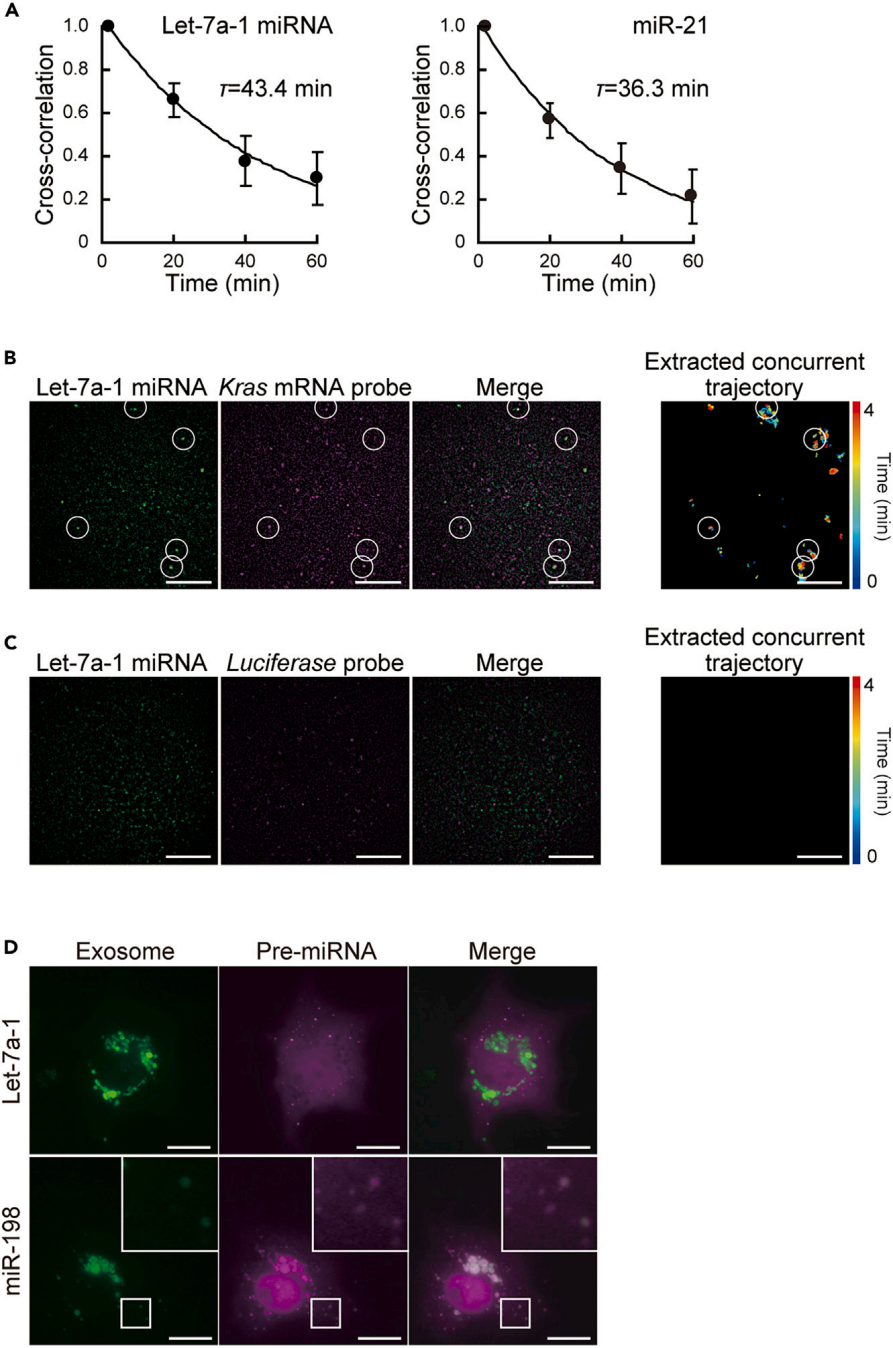
Confirmation of maturation of introduced miRNAs in single living cells (A) Time courses of the normalized amplitude of fluorescence cross-correlation functions (FCFs) of fluorescently labeled guide and passenger strands of let-7a-1 pre-miRNA (left) and pre-miRNA of miR-21 (right) in the cytoplasm. The decay curves of FCFs were approximated with an exponential decay with time constants of 43.4 min (let-7a-1) and 36.3 min (miR-21). Error bars represent SD (n = 5 cells). (B) Colocalization of let-7a-1 pre-miRNA (left), its target *Kras* mRNA antisense probes (middle), and their merged (right) image. Trajectories of extracted foci in which (pre-)miRNA and mRNA antisense probe(s) co-localized are shown (far right). White circles indicate the example of the colocalization of let-7a-1 (pre-) miRNA and *Kras* mRNA. (C) Fluorescence images of let-7a-1 pre-miRNA (guide strand was labeled with 2MeSiR, left) and *Luciferase* mRNA antisense probe (labeled with Cy3B, middle), and their merged (right) image. No co-localized focus was extracted (far right). (B and C) Representative single-frame images of let-7a-1 and mRNA probes at a given time are shown. White scale bars indicate 10 μm. (D) Localization of the specific miRNA in exosome. Epi-fluorescence images of exosome visualized with CD63-GFP (left), pre-miRNAs (middle), and their merged images (right). miRNA-198 localized in exosomes, while let-7a-1 miRNA did not. The regions shown in the squares in the lower panels are enlarged in insets above them to the right in the panels. White scale bars indicate 20 μm.

Taking advantage of the aforementioned stability of miRNA in the cytoplasm visualized by introducing pre-miRNA, we observed the maturation of miRNAs by quantitative single-molecule sensitive analysis to examine whether miRNAs visualized by pre-miRNA introduction are incorporated into the endogenous pathway. First, we focused on the dissociation of guide and passenger strands, a critical step in miRNA maturation. By using FCCS,^30^ a quantitative analysis of molecular diffusion, this dissociation can be tracked with high sensitivity at the single-cell level. After introducing let-7a-1 pre-miRNA comprising Alexa 488-labeled guide strand and Cy5-labeled passenger strand, cross-correlation functions (CCFs) between the two strands were acquired by FCCS. The resultant CCFs of let-7a-1 pre-miRNA showed that the cross-correlation of pre-miRNA gradually decreased as time passed and the time constant was estimated to be about 40 min (Figure 2A, left). Furthermore, the CCFs of pre-miRNA miR-21, which was fluorescently labeled in the same way, showed a time-dependent decrease in the cross-correlation between the two strands (time constant 36 min, Figure 2A, right). This indicates that the introduced pre-miRNAs examined here were endogenously processed and the duplex was unwound with a time constant of approximately 40 min.

To directly observe whether the introduced pre-miRNA binds to its target mRNA following maturation, we used a single-molecule imaging method that uses stochastic blinking of specific fluorophores: Cy3B, 2MeSiR,^31^ and Cy5. Tracking single molecules requires inducing the majority of dye to adopt a dark state and detecting only a small number of molecules. The blinking dyes used here are better suited for long-term tracking than dyes such as Alexa 488 and Cy3, which only irreversibly quench by photobleaching. First, Cy3B-labeled antisense probes^32^ targeting *Kras* mRNA were introduced for imaging endogenous *Kras* mRNA, the target of let7a-1 miRNA, allowing them to bind to *Kras* mRNA. Then, 2MeSiR-labeled let-7a-1 pre-miRNA was introduced into the same cells. In 60 min, two-color simultaneous single-molecule imaging of 2MeSiR and Cy3B was performed. The co-localization of the two fluorescent colors was observed (Figure 2B). Furthermore, analysis of these images revealed that, while co-localizing, the two fluorescent colors showed the same trajectory (Figure 2B). When the antisense probe of *Luciferase* mRNA was used as a control, no co-localization of the two colors was detected (Figure 2C). The above results revealed the sequence-specific concurrent movement of let-7a-1 miRNA with its target mRNA, suggesting that a fraction of the introduced let-7a-1 pre-miRNA associates with the target mRNA in cells. To quantitatively capture the specific binding of the introduced let-7a-1 pre-miRNA to the target mRNA, we calculated the percentage of all miRNAs detected during the observation that were co-localized with the target mRNA. As a result, 19% of the let-7a-1 miRNAs (59811 particles) detected during the 8-min observation co-localized with *Kras* mRNA (11364 particles). In contrast, only 2.3% of the let-7a-1 miRNAs (61693 particles) detected in the control experiment co-localized with the luciferase antisense probe (1452 particles). This co-localization ratio (19%) is considered reasonable because let-7a-1 miRNA has multiple targets in addition to *Kras* mRNA.

We studied the intracellular delivery of pre-miRNA 60 min after introduction into cells. We first investigated the encapsulation of miRNA into exosomes. Some specific miRNAs including miR-198 have been reported to be sorted into exosomes in a sequence-dependent manner.^16^ Upon the introduction of fluorescently labeled pre-miRNA of miR-198 into cells whose exosomes were visualized with CD-63-GFP, it was shown to colocalize with exosomes at 60 min after microinjection, while let-7a-1 miRNA did not accumulate in specific subcellular compartments, but was spread throughout the cytoplasm (Figure 2D). The results recapitulated previous findings regarding the type-dependent localization of miRNAs to exosomes. To further investigate the delivery to the nucleus, we next examined the nuclear signal of the introduced let-7a-1 pre-miRNA after 60 min. The results showing more nuclear accumulation of the guide strand compared to the passenger strand (Figure S4) are consistent with studies of nuclear miRNAs.^12^^,^^13^^,^^14^

Taken together, these results indicate that the dynamic intracellular behavior of the miRNA visualized in this study is very consistent with that of endogenous miRNA. Furthermore, the ability of the fluorescently labeled let-7a-1 pre-miRNA used in this study to inhibit translation was also confirmed (Figure S5),^33^ which is in line with previous reports demonstrating this capability of fluorescently labeled miRNA duplex^24^ and unlabeled pre-miRNA.^26^^,^^27^ In conclusion, it was confirmed that part of the fluorescent pre-miRNA introduced into living cells is incorporated into the endogenous miRNA maturation process, such as RISC formation, thereby exhibiting miRNA function.

### SPT reveals cytoskeleton-dependent directed movement of miRNAs

**Figure 3:**
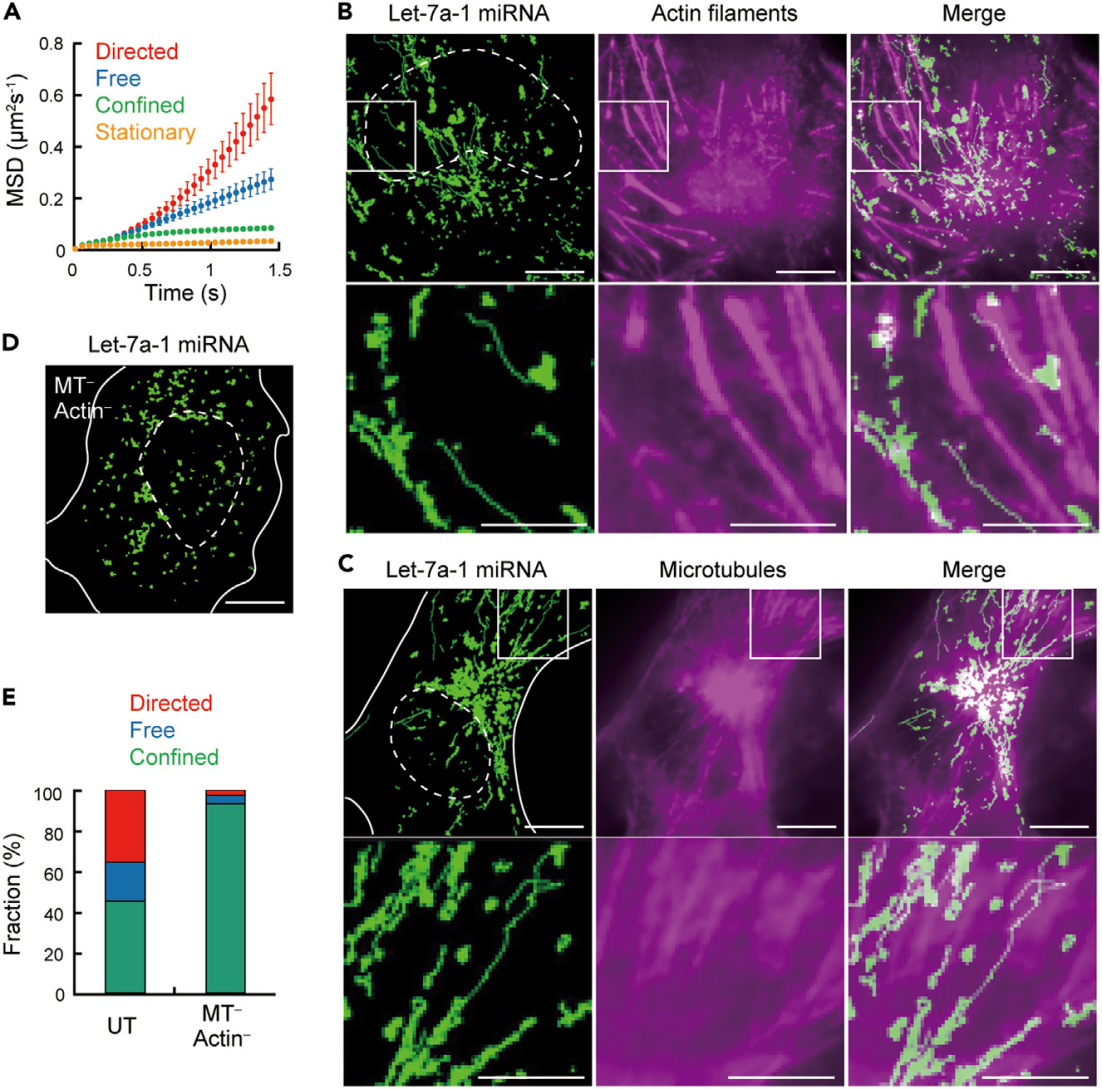
Single-particle tracking of miRNAs visualized by the introduction of pre-miRNA reveals cytoskeleton-dependent directed movement of miRNA in living cells (A) Mean square displacement (MSD) analysis of single miRNA particles. Different modes of molecular dynamics were observed: directed (red, n = 778), free (blue, n = 417), confined (green, n = 984), and stationary (yellow, n = 4451). Bars represent SD. (B) Tracking of miRNA particles on actin filaments. Trajectories of single let-7a-1 miRNA particles (left), actin filaments (middle), and their merged image (right). The images in the lower panels are enlargements of areas shown in the upper panels. Scale bars, 10 μm (top), 5 μm (bottom). (C) Tracking of miRNA particles on microtubules. Trajectories of single let-7a-1 miRNA particles (left), microtubules (middle), and their merged image (right). The images in the lower panels are enlargements of areas shown in the upper panels. Scale bars, 10 μm (top), 5 μm (bottom). (D) Disruption of cytoskeletal components impairs directed movement of let-7a-1 miRNA. Trajectories of let-7a-1 miRNA in a living cell in which actin filaments (Actin) and microtubules (MT) were disrupted by cytochalasin D and nocodazole, respectively, are shown. Scale bar, 10 μm. (E) Fractions of three diffusion modes of mobile miRNA: directed (red), free (blue), and confined (green). Notably, the components excluding stationary are shown here. Untreated cells (UT, left) and cells in which microtubules (MT) and actin filaments (Actin) were disrupted (MT^–^/Actin^–^, right) are shown. Dotted and solid lines indicate the nucleus and the cell periphery, respectively.

Next, 60 min after the introduction of Cy5-labeled pre-miRNAs (let-7a-1, miR21 and miR198), we performed SPT to observe miRNA behavior. The direct visualization of miRNA particles showed various modes of movement within living cells. Time-dependent mean square displacement (MSD-t) plots allow quantitative classification of patterns of movement (i.e., stationary, restricted diffusion, free diffusion, and directed movement). The obtained MSD-t plots of the introduced miRNA identified the four patterns of movement, including directed movement (Figure 3A). As miRNAs form RISC and then further bind to their target mRNAs, their molecular diffusion is considered to be dependent on the overall size of the complex. Therefore, we believe the effect of sequence-dependent secondary structures of individual miRNAs would be small and did not consider secondary structures of the miRNAs themselves in the interpretation of miRNA trafficking data.

On the basis of the results of observed directed movement of miRNA and the fact that the miRNA-binding protein Ago interacts with cytoskeletal components such as actin filaments and tubulin^34^^,^^35^ and that pre-miRNAs are actively trafficked along axon microtubules in retinal ganglion cells,^36^ directed movement of miRNAs is expected to be related to cytoskeletal components, including actin filaments and microtubules. Here, we visualized the trajectory of a single miRNA particle and the cytoskeleton simultaneously by performing alternate imaging for miRNA single particle trajectories and for cytoskeletal distributions. In this case, we used a 50 ms exposure time to match the temporal resolution of the SPT, leading to a relatively poor signal-to-noise ratio for fluorescent imaging of the cytoskeletons with this exposure time. First, we visualized let-7a-1 miRNA at the single-particle level in cells in which actin filaments were visualized. As shown in Figures 3B and Video S1, several miRNA particles were observed to migrate along actin filaments. The velocity of the bright spots showing directed movement was several microns per second. This velocity matched that of intracellular motor proteins,^37^ suggesting that miRNAs underwent motor protein-mediated movement on actin filaments. Second, the visualization of microtubules showed that some miRNAs moved along microtubules (Figures 3C and Video S2). These phenomena were observed in all cells analyzed, and other miRNAs (miR-21 and miR-198) also showed directed movement in cells (Figure S6). In contrast, in cells in which both actin filaments and microtubules were disrupted, miRNA particles did not show directed movement (Figures 3D and 3E, and Video S3). These results confirmed that miRNAs investigated in this study moved in a cytoskeleton-dependent manner. It should be noted that because cytoskeletons are dynamic, superimposing a photograph of cytoskeletal components at a given moment with the trajectories of miRNAs may not be completely co-localized. However, even taking this into account, we notice that the trajectories of miRNAs visualized together with the cytoskeletons are distributed not only on the cytoskeletons themselves but also in regions other than the cytoskeletons.

Video S1. Tracking of miRNA particles along actin filaments in living cells, related to Figure 3 Video of Cy5-labeled let-7a-1 miRNA particles (red, 19 times faster than real time) is merged with averaged image of actin filaments (green).

Video S2. Tracking of miRNA particles along microtubules in living cells, related to Figure 3 Video of Cy5-labeled let-7a-1 miRNA particles (red, 19 times faster than real time) is merged with averaged image of microtubules (green).

Video S3. Tracking of miRNA particles in cytoskeleton-disrupted cells, related to Figure 3 Video of Cy5-labeled let-7a-1 miRNA particles in living COS7 cells in which both actin filaments and microtubules are destroyed. The video runs 19 times faster than real time.

### Trafficking of miRNAs on cytoskeletal components associated with the perinuclear region

**Figure 4:**
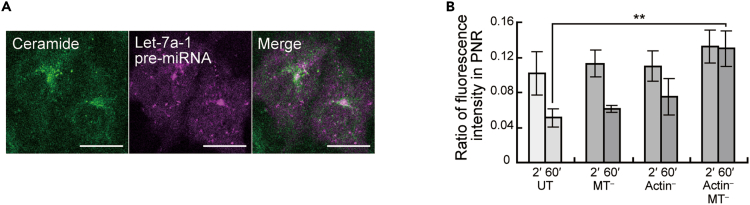
Trafficking of miRNAs on cytoskeleton associated with the perinuclear region (A) Accumulation of introduced miRNA in the perinuclear region (PNR). Confocal fluorescence images of BODYPY-TR-labeled ceramide (left), let-7a-1 pre-miRNA (Cy5-labeled guide strand, middle), and their merged image (right). (B) Cytoskeleton-dependent outflow of miRNA from the perinuclear region (PNR). The proportion of fluorescence intensity of let-7a-1 pre-miRNA (Cy3-labeled guide strand) in the PNR relative to that of the whole cell at 2 and 60 min after introduction into the cytoplasm of living cells. UT, MT^–^, Actin^–^, and MT^–^/Actin^–^ indicate untreated cells, cells with microtubule disruption, cells with actin filament disruption, and cells with both microtubule and actin filament disruption, respectively. ∗∗p < 0.01; *t*-test. Error bars represent SD [n = 6 cells (UT), 3 cells (MT^–^), 4 cells (Actin^–^), 4 cells (MT^–^/Actin^–^)].

In all cells observed, most of the miRNAs that showed directed movement accumulated in and spread from the perinuclear region in a radiating manner (Figures 3B, 3C, and S6). This perinuclear region where let-7a-1 miRNA localized corresponded to the location of the Golgi apparatus (Figure 4A). This result is consistent with the findings that key components of RISC and miRNA accumulate in the ER–Golgi apparatus in the perinuclear region to repress mRNA translation.^12^^,^^38^^,^^39^ This accumulation of the miRNA in the perinuclear region increased when both actin filaments and microtubules were disrupted (Figure 4B). This suggests that these cytoskeletal components facilitate the trafficking of let-7a-1 miRNA from the perinuclear region. Collectively, these results suggest a model in which cytoskeleton-dependent miRNA movement is responsible for the trafficking of miRNA from the perinuclear region to other intracellular regions.

## Discussion

In this study, we first developed an efficient method to visualize miRNA in living cells by using the endogenous biosynthesis process of the introduced fluorescently labeled pre-miRNA precursors. Compared with the conventional method of introducing fluorescently labeled miRNA duplexes, this method was suggested to be more effective in observing miRNAs in living cells due to its higher retention in cells. Because miRNA visualization by the direct introduction of fluorescent pre-miRNA into cells is performed at the single-cell level, this method is not suitable for investigating molecular mechanisms related to miRNA by biochemical methods such as electrophoresis and immunoprecipitation. Therefore, in this study, we employed single-molecule sensitive microscopy to confirm that the observed signals recapitulate miRNA processing. Furthermore, by tracking the detailed behavior of the introduced miRNAs with SPT, we found that the miRNAs investigated in this study exhibited cytoskeleton-dependent trafficking, which is associated with organelles such as the Golgi apparatus and ER.

The basic properties of miRNA in cells observed with fluorescent pre-miRNA, such as its ability to repress translation and accumulation in PB and the nucleus, are consistent with previous studies visualizing miRNA with fluorescent miRNA duplex.^10^^,^^11^^,^^24^^,^^25^ In addition, the directed movement of miRNA detected in this study is consistent with the biased motion observed in the previous work using miRNA duplex.^24^ Meanwhile, the fluorescent pre-miRNA employed in this study showed higher stability in living cells than the fluorescent miRNA duplex, indicating that the introduced pre-miRNA is more efficient at being incorporated into stable complexes. The percentage of remaining guide strands of the introduced pre-miRNA (approximately 50–60%, Figure 1B) indicates its higher stability in cells, compared to approximately 10–20% for the introduced miRNA duplex, which is located downstream of miRNA biogenesis. Considering that duplexes that are not covalently bonded can easily become unstable single-stranded miRNAs in living cells, this difference may be attributable to the structural feature of pre-miRNA whose passenger strand does not spontaneously dissociate unless its loop is degraded. The presence of a loop in the pre-miRNA may also increase the opportunity for interaction with factors that are more responsible for biosynthesis compared to the simpler structures, duplex and single stranded miRNA. This may protect the pre-miRNA from degradation until the complex with Dicer is delivered to the Ago proteins. Indeed, a large number of pre-miRNAs served as substrates for biosynthetic reactions such as passenger strand dissociation (Figure 2). Thus, we believe that the introduced pre-miRNAs are highly efficient for incorporation into the complexes responsible for endogenous biosynthetic pathways. These results suggest that the observed behavior of pre-miRNAs (Figures 3 and 4) reflects the nature of endogenous miRNAs. Therefore, this method is suitable for detailed analysis of miRNA behavior in cells. Because of this advantage, the high fraction (12% of all visualized miRNAs and 36% of mobile miRNAs) of miRNAs visualized by this method that showed directed movement led to the discovery of cytoskeleton-associated miRNA trafficking. In the future, the introduction of precursors further upstream in the biosynthetic process can be considered (e.g., injection of fluorescently labeled pri-miRNA into the nucleus), but it will be necessary to investigate the state and function of each introduced precursor, because the factors interacting with each precursor and its distribution within the cell are also different.

The time constant for the dissociation of the passenger strand in the maturation of the introduced pre-miRNAs, including loop removal (∼40 min), was longer than the result using miRNA duplex (∼20 min).^25^ This suggests that this method, which uses the intracellular introduction of pre-miRNA, a more upstream precursor of the biogenesis pathway, is suitable for understanding longer endogenous maturation processes (e.g., incorporation into the RISC-loading complex, removal of the loop structure by Dicer, and separation of the miRNA duplex by RISC) that are interconnected with each other.^40^^,^^41^^,^^42^ By tracking miRNA from the pre-miRNA stage, this technique will also help to elucidate the miRNA functions and regulatory mechanisms coupled with the maturation processes that pre-miRNA undergo, an area that has received recent attention.^43^^,^^44^

A new biological insight into miRNA dynamics revealed in this study was cytoskeleton-dependent trafficking. Because the miRNAs visualized by this method is considered to include both immature and mature miRNAs, it is unclear which part of the various maturation stages of miRNA would interact with the cytoskeletons, while the cytoskeletal components may be involved in diverse miRNA functions (e.g., miRNA maturation, miRNA-driven translation repression, and/or miRNA-mediated cell-to-cell communication). In addition, our direct observation of the miRNAs revealed that they exhibit directed movement on cytoskeletal components and accumulate and diffuse into the perinuclear region in a cytoskeleton-dependent manner. The results showing that disruption of the cytoskeleton reduced the rates of directed movement and free diffusion and made restricted diffusion dominant suggest that cytoskeleton-dependent miRNA trafficking allows the intracellular distribution of miRNA complexes that cannot travel without the cytoskeleton. The trajectories of miRNAs visualized together with the cytoskeleton are distributed not only on the cytoskeleton, but also in regions outside of the cytoskeleton. Considering these results together with those of cytoskeletal disruption, we can speculate both forms of intracellular miRNA transport, one on the cytoskeleton and the other dependent on other structures and flows associated with the cytoskeletons. This study provides experimental evidence for a recently proposed model of microtubule-mediated trafficking of miRNA within cells.^45^ Previous studies have shown that mRNA forms a complex with miRNAs as soon as it is exported from the nucleus, leading to translation repression,^46^^,^^47^ and that mRNA is trafficked on the cytoskeleton.^48^ Combining these facts with our own, miRNA may encounter mRNA in the ER in the perinuclear region and repress translational activity via RISC formation, and then be efficiently and selectively be distributed within the cell by the cytoskeleton. It thereby contributes to local translation control not only in the ER but also at any location in the cell. Furthermore, this study may have achieved direct observation of miRNA trafficking on the cytoskeleton being involved in the gap junction-mediated cell-to-cell transport of miRNA, as proposed by Lemcke et al.^49^ Collectively, we speculate that this unique cytoskeleton-dependent behavior of miRNA may contribute to the localization of miRNA to intracellular organelles and distal compartments in polarized cells^36^ and its export from cells.

Our technique for visualizing miRNA in cells via the introduction of fluorescently labeled pre-miRNA is applicable to all types of miRNAs and cells. A comprehensive study of various miRNAs will demonstrate the universality of dynamic miRNA behavior (e.g., cytoskeleton-dependent miRNA trafficking) in living cells. In addition, owing to the high intracellular retention of introduced pre-miRNA, this method for visualizing miRNA in cells should contribute to the improvement of various miRNA imaging methods that have recently been developed by introducing exogenous miRNA precursors and derivatives,^11^^,^^24^^,^^25^^,^^50^^,^^51^ and to the combination with single-molecule imaging of mRNA and miRNA-related proteins to study post-transcriptional control.^46^ By using this technique to directly observe miRNA processing, localization, and trafficking in various pathological cells, including cancer cells, we can expect breakthroughs regarding the mystery of miRNA aberrations observed in a wide range of diseases.^3^ Although we have revealed that introduced miRNA moves along cytoskeletal components, the molecular mechanisms behind the association between miRNA and the cytoskeleton and its differences (if any) from the dynamic nature (including its stability and processing) of endogenous miRNA are still unclear. In the search for these mechanisms, important milestones include the findings that the RISC-loading complex and key components of RISC, such as Dicer and AGO, interact with cytoskeletal components.^34^^,^^35^ In the future, direct observation of miRNA in various cellular environments, including cells with manipulation of these miRNA-associating molecules and cytoskeleton-related molecules, will be necessary to clarify how miRNA is actively transported and dynamically interacts with organelles. In this study, we assumed that the changes in miRNA motility and intracellular delivery caused by cytoskeletal disruption are directly due to the inhibition of transport on the cytoskeleton, but it is also possible that changes in the intracellular environments governed by the cytoskeletons may indirectly inhibit miRNA transport and association with mRNA. Therefore, future research into the mechanism of miRNA transport should include not only the manipulation of the cytoskeleton and its behavior, but also various related factors. These studies will deepen our understanding of the mechanisms and physiological significance of cytoskeleton-dependent subcellular localization, organelle interactions, and cell-to-cell transport of miRNA.

### Limitations of the study

In this study, we observed the trafficking of three miRNA species in COS7 cells dependent on the cytoskeleton. It remains unclear whether such a phenomenon occurs in other cell types or miRNA species, and verification using our method is necessary. Additionally, the extent to which the miRNA dynamics (maturation and trafficking) measured here accurately reflect the properties of endogenous miRNAs requires further verification. For example, fluorescent labeling of exogeneous miRNAs may alter the rate and efficiency of miRNA processing, which would imply the measured dynamics may not fully reflect the properties of native miRNAs. To examine the dynamics of native miRNAs, it is necessary to develop further visualization techniques.

## STAR★Methods

### Key resources table

**Table:** 

REAGENT or RESOURCE	SOURCE	IDENTIFIER
**Chemicals, peptides, and recombinant proteins**		

DEPC-treated water	Thermo Fisher	Cat#AM9906
T4 RNA Ligase 1	New England BioLabs	Cat#M0204S
FuGENE6	Promega	Cat#E2691
BODIPY TR ceramide complexed to BSA	Thermo Fisher	Cat#B34400
Phalloidin, tetramethylrhodamine B isothiocyanate	Sigma Aldrich	Cat#P1951
CellLight Tubulin-GFP, BacMam 2.0	Thermo Fisher	Cat#C10613
Cy3B NHS-ester	GE Healthcare Life Sciences	N/A
Streptavidin	Thermo Fisher	Cat#21125
Cytochalasin D	Wako	Cat#034-25881
Nocodazole	Sigma Aldrich	Cat#M1404

**Critical commercial assays**		

Small RNA Gel Extraction Kit	TaKaRa Bio	Cat#9106
ZR small-RNA™ PAGE Recovery Kit	Zymo Research	Cat# R1070

**Experimental models: Cell lines**		

COS7	Riken Cell Bank	Cat#RCB0539
HeLa	Riken Cell Bank	Cat#RCB0007

**Oligonucleotides**		

Fluorescently labeled miRNA precursors (Table S1)	Japan Bio Services	https://www.jbios.co.jp
Fluorescently labeled antisense 2'-*O*-methyl RNA probes (Table S2)	Japan Bio Services	https://www.jbios.co.jp

**Recombinant DNA**		

CD-63-GFP	System Biosciences	Cat#CYTO120

**Software and algorithms**		

AQUA-Lite ver. 10	Hamamatsu Photonics	https://www.hamamatsu.com
LAS-AF WPF	Leica Microsystems	https://www.leica-microsystems.com
SymPhoTime	PicoQuant	https://www.picoquant.com/products/category/software
KaleidaGraph	Synergy Software	https://www.synergy.com
TrackMate v2.0.4	Tinevez, J.Y et al.^52^	https://doi.org/10.1016/j.ymeth.2016.09.016
ImageJ/Fiji	NIH	https://imagej.net

### Resource availability

#### Lead contact

Further information and requests for resources and reagents should be directed to and will be fulfilled by the Lead Contact, Dr. Kohki Okabe (okabe@mol.f.u-tokyo.ac.jp).

#### Materials availability

This study did not generate new unique reagents.

#### Data and code availability

Data

• All raw data or information related to image analysis presented in this paper is available from the lead contact upon request.

Code

• This paper does not report original code.
• Any additional information required to reanalyze the data reported in this paper is available from the lead contact upon request.

### Experimental model and study participant details

#### Cell culture

In this study, in addition to HeLa cells from human (Figure S1), COS7 cells from monkey, which are suitable for microinjection, were used. The miRNA and target mRNA sequences examined are conserved among mammals including these species.^53^ COS7 cells and HeLa cells (Riken, Wako, Japan) were cultured in Dulbecco’s Modified Eagle’s Medium (DMEM) with 10% fetal bovine serum supplemented with penicillin–streptomycin, L-glutamine, sodium pyruvate, and nonessential amino acids at 37°C in 5% CO_2_. For live-cell imaging, cells were cultured in 35-mm glass-bottomed dishes (AGC Techno Glass, Yoshida, Japan) and the medium was replaced by phenol red-free culture medium containing HEPES buffer (2 mL) before live-cell imaging. All solutions were from Thermo Fisher Scientific.

### Method details

#### Preparation of fluorescent microRNA precursors

All single-stranded RNAs (purified by HPLC; Table S1) including those labeled with Alexa488, Cy3, and Cy5 were purchased from Japan Bio Services (Asaka, Japan), followed by the preparation of miRNA duplex and pre-miRNA as described below. All synthesized miRNA precursors were dissolved in DEPC-treated water (Thermo Fisher Scientific, Waltham, MA).

#### MiRNA duplex

Two single-stranded miRNAs the 3′ ends of which were labeled with either Cy3 (guide strand) or Cy5 (passenger strand) were mixed and heated to 80°C, followed by gradual cooling to 4°C to allow hybridization. The resultant duplexes were confirmed by 15% polyacrylamide gel electrophoresis.

#### Pre-miRNA

The amino moiety in the middle of pre-miRNA was reacted with *N*-hydroxysuccinimide (NHS) ester of 2MeSiR.^31^ Fluorescent strands of pre-miRNA were ligated with passenger sequences using T4 RNA Ligase 1 (New England BioLabs, Ipswich, MA) for 16 h at 22°C. After precipitation with ethanol, the solutions were subjected to 15% polyacrylamide gel electrophoresis (containing 1 M urea). The bands corresponding to pre-miRNAs were cut out and extracted with small RNA Gel Extraction Kit (TaKaRa Bio, Kusatsu, Japan) or ZR small-RNA™ PAGE Recovery Kit (Zymo Research, Irvine, CA).

#### Microinjection of fluorescent probes into living cells

Microinjection of miRNA and antisense probes into living cells was performed with Femtojet (Eppendorf, Hamburg, Germany), controlled by a micromanipulator (Eppendorf). The RNA solutions were dissolved in an aqueous solution containing 80 mM KCl, 10 mM KH_2_PO_4_–K_2_HPO_4_ (pH 7.2), and 4 mM NaCl to a concentration of 1 μM (pre-miRNA) or 3 μM (single-stranded miRNA, duplex miRNA, and antisense probes). The solution was filtered using an Ultrafree-MC (Millipore, Burlington, MA) and microinjected into the cytoplasm of cells using a glass capillary needle (Femtotips II; Eppendorf) by pressing the needle tip against the center of the cytoplasm while exhaling at compensation pressure of 20–50 hPa. The volume of injected solution was estimated to be 2 fL (this amount varies from cell to cell).

#### Fluorescence imaging of the cells

##### Epi-fluorescence imaging

Live-cell epi-fluorescence imaging (Figures 1A, 1B, 2D, and S1–S5) was performed on an IX70 inverted microscope (Olympus, Tokyo, Japan) equipped with an objective lens (60×, UplanApo N.A. 1.40; Olympus). The temperature of the culture medium was maintained at 37°C by controlling with a stage and a microscope objective lens heater with a controller (MI-IBC; Olympus). A cooled CCD camera (ORCA-ER; Hamamatsu Photonics, Hamamatsu, Japan) was used for the acquisition of cell images. The fluorescence images of EGFP and Alexa488 were taken using a sapphire laser (488-nm Model 488-30 CDRH; Coherent), a dichroic mirror (DM505; Olympus), and an emission filter (BA515-550; Chroma Technology, Yokohama Japan). The fluorescence images of Cy3 were taken with a green solid-state laser (532 nm Compass 315M-100; Coherent, Santa Clara, CA), a dichroic mirror (Q565LP; Olympus), and an emission filter (HQ610/75M; Chroma Technology). The fluorescence images of Cy5 and RFP were taken using a red He–Ne laser (633-nm GLS5360; Showa Optronics), a dichroic mirror (660LP; Olympus), and an emission filter (700/75M; Chroma Technology). Fluorescence images were quantitatively analyzed using AQUA-Lite ver. 10 (Hamamatsu Photonics) at various intervals after microinjection.

#### Confocal fluorescence imaging

Confocal fluorescence imaging (Figures 1C and 4A) was performed on a TCS SP8 confocal laser-scanning microscope (Leica Microsystems, Wetzlar, Germany). The fluorescence was captured through objective lenses (63×, HC PL APO CS2 1.20 water, and an HCX PL APO Ibd.BL 1.4 N.A.1.4 oil; Leica) with 1–9 zoom factors in 1024×1024-pixel format. The temperature of the culture medium was maintained at 37°C by controlling with a stage and a microscope objective lens heater with a controller (INUBSF-ZILCS; Tokai Hit, Fijinomiya, Japan). The fluorescence images of EGFP, Bodipy, and Cy5 were excited with 488-, 552-, and 638-nm solid lasers (Leica), respectively, and captured with a PMT and HyD detector (Leica). The images were merged with LAS-AF WPF software (Leica).

#### Highly inclined and laminated optical sheet (HILO) fluorescence imaging

HILO fluorescence imaging (Figures 2B, 2C, 3A, 3B, 3C, 3D, 3E, and S6) was performed on an N-STORM inverted fluorescence microscope (Nikon) with an oil-immersion objective (APO TIRF 100× N.A., 1.49; Nikon, Tokyo, Japan). The temperature of the culture medium was maintained at 37°C by controlling with a stage and a microscope objective lens heater with a controller (INUH-TIZSH-F1; Tokai Hit). The fluorescence images of tetramethylrhodamine B and Cy3B were excited with a 561-nm solid laser (Sapphire 561 LP; Coherent), EGFP was excited with a 488-nm solid laser (Sapphire 488 LP; Coherent), Cy5 and 2MeSiR were excited with a 647-nm solid laser (2RU VFL P 200 647; MPB Communications, Pointe-Claire, Canada), and captured with a back-illuminated EMCCD camera (iXon3 Ultra897; Andor Technology, Belfast, UK). The image analysis was performed with ImageJ/Fiji.

#### Visualization of organelles

Processing body (PB) and exosome were visualized with EGFP-Dcp1 and CD-63-GFP (System Biosciences, Palo Alto, CA) plasmids, respectively, which were transfected in COS7 cells using FuGENE6 (Promega, Madison, WI) following the manufacturer’s protocol. The Golgi apparatus was visualized with BODIPY TR ceramide complexed to BSA (Thermo Fisher Scientific). In cells in which either exosome or Golgi apparatus was visualized, fluorescence images of miRNA and organelles were taken 60 min or more after pre-miRNAs were microinjected. Actin was visualized with phalloidin, tetramethylrhodamine B isothiocyanate (Sigma Aldrich, St. Louis, MO), which was resuspended in DEPC-treated water (Thermo Fisher Scientific) and microinjected into COS7 cells. Microtubules were visualized with CellLight Tubulin-GFP, BacMam 2.0 (Thermo Fisher Scientific).

#### Confirmation of translational repression by fluorescent miRNAs

Two plasmids of the GFP gene containing the target sequence of let-7a-1 and RFP that did not contain a miRNA target^33^ were transfected using FuGENE 6 Transfection Reagent (Promega) into COS7 cells in which Cy3-labeled let-7a-1 pre-miRNA had been microinjected, following the manufacturer’s protocol. The fluorescence of GFP and RFP was quantified after 2 days of incubation.

#### Fluorescence (cross)correlation spectroscopy (FCS/FCCS)

Fluorescence correlation spectroscopy (FCS) and fluorescence cross-correlation spectroscopy (FCCS) (Figure 2A) were conducted on a confocal laser-scanning fluorescence microscope (TCS SP8; Leica) equipped with a single-molecule detector unit (PicoQuant, Berlin Germany). The fluorescence of Alexa488 and Cy5 was captured through an objective lens (63×, HC PL APO CS2 1.20 N.A. water; Leica), a dichroic mirror (BS 620; Leica), and emission filters (BP500-550 and BP 647-703; Leica). Five seconds of fluorescence fluctuation was recorded, allowing the calculation of auto- and cross-correlation using software (SymPhoTime, PicoQuant). The obtained fluorescence autocorrelation (FAC) and fluorescence cross-correlation (FCC) between 0.01 and 813 ms were approximated with an autocorrelation function (Equation 1), having one or two components, with SymPhoTime (PicoQuant):

Equation 1 $$G\left(\tau \right)-1=\frac{1}{N}\times \left(\frac{1}{1+\tau /\tau_{1}}\right){\left(\frac{1}{1+{\left(1/\kappa \right)}^{2}\left(\tau /\tau_{1}\right)}\right)}^{\frac{1}{2}}$$

where *N* represents the number of fluorescent dyes in the confocal volume, *τ*_1_ represents the diffusion time, and *κ* represents the structure parameter (10–15 in this experiment).

The time-dependent change of FCCS was obtained from the same cells and was normalized at the first observation (2 min after microinjection) and approximated with an exponential function using KaleidaGraph software (Synergy Software, Reading, PA) to yield the time constant of the dissociation of miRNA duplexes.

#### Single-particle tracking of miRNAs and mRNA

Single-particle fluorescence imaging of Cy3B, Cy5, and 2MeSiR was performed by HILO microscopy (described above) on an N-STORM inverted fluorescence microscope (Nikon). In the case of imaging Cy5, cells were cultured on a medium containing glucose (2.25 mg/mL), catalase (25 U/mL), glucose oxidase (25 U/mL), and Trolox (0.5 mM); no obvious cell damage was observed during observation (about 20 min) in this oxygen scavenging system. First, strong lasers (100% output power) were applied for 20 s to induce most of the fluorescent molecules to adopt a dark state. Then, 20,000 frames were acquired with a weak laser (5% output power) at a frame rate of 20 Hz. The focus was adjusted using a Perfect Focus System. Single-particle tracking was performed with TrackMate^52^ v2.0.4. For the detection of particles, the DoG detector was used with a diameter of 0.5 μm and a quality parameter of 50. To establish trajectories from the detected points, the nearest neighbor algorithm was employed, where the maximum distance of travel between any two points in consecutive frames was set to 0.4 μm. Trajectories over 2.8 s were extracted for further analysis. To detect *Kras* mRNA in COS7 cells, we prepared 2′-*O*-methyl RNA (2′OMe RNA) antisense oligonucleotides containing an amino moiety (Table S2; Japan Bio Services). The 2′OMe RNA oligonucleotides were labeled with Cy3B (GE Healthcare Life Sciences, Chicago, IL) via an NHS-ester reaction to primary amines. Furthermore, to avoid accumulation of the probe in the nucleus, the probe was conjugated to streptavidin (Thermo Fisher Scientific) via biotin attached to the probe. By adding an excess amount (eight times the molar concentration ratio) of streptavidin, which has four biotin-binding sites, we prevented the oligomerization of the probe to the streptavidin.^32^ The three kinds of oligonucleotides (3 μM) were mixed and microinjected into living COS7 cells, followed by single-molecule imaging.

#### MSD analysis and categorization of tracks

MSD analysis and categorization of tracks were performed in a manner similar to that reported previously.^54^ MSD for each trajectory was calculated by Equation 2:

Equation 2 $$\text{MSD}\left(t=n\cdot \Delta t\right)=\frac{\sum_{i=1}^{N-n}\left[{\left(x_{i+n}-x_{i}\right)}^{2}+{\left(y_{i+n}-y_{i}\right)}^{2}\right]}{N-n}$$

where *N* is the time window for analysis, *n* is the frame number to be analyzed, Δ*t* is the time resolution (50 ms), and *x*_i_ and *y*_i_ are the coordinates of the detected points at frame *i*. Categorization of tracks was performed as follows. For each trajectory, MSD was calculated using 55 frames. First, trajectories with a diffusion coefficient *D* of 0.01 μm^2^/s or less, calculated from the first 2–11 frames, were classified as “immobile” and the others (those with a diffusion coefficient greater than 0.01 μm^2^/s) were classified as “mobile.” The MSDs of 2–30 frames of trajectories classified as mobile were fitted with the equation of confined motion (Equation 3: initial value: *R*_conf_ = 0.3, *τ* = 20), and *τ* was calculated.

Equation 3 $$\text{MSD}\left(t\right)=\frac{4R_{\text{conf}}^{2}}{3}\left(1-e^{-t/\tau }\right)$$

Obtained *τ* values of 30 frames or fewer were classified as confined, while those of greater than 30 frames were classified as diffusive or directed. The MSD (between 2 and 30 frames) classified as diffusive or directed were further fitted with a quadratic function (MSD = a*t*^2^: initial value: a = 0.2) and classified as directed if the obtained slope a was greater than 4D×1.2, and diffusive if it was smaller.

#### Disruption of cytoskeletal components

The disruption of actin filaments and microtubules in living cells was conducted by incubation with cytochalasin D (0.1 μg/mL; Wako) for 3 h and nocodazole (100 ng/mL; Sigma Aldrich) for 12 h, respectively.

### Quantification and statistical analysis

#### Image analysis

Epi-fluorescence were quantitatively analyzed using AQUA-Lite ver. 10 (Hamamatsu Photonics). Confocal fluorescence images were merged with LAS-AF WPF software (Leica). HILO fluorescence images were analyzed using ImageJ/Fiji.

#### Statistical analysis

Statistical tests were performed using Excel software. Mann-Whitney U test and t-test was used for comparisons. ^∗∗^*p* ≤ 0.01.
