# Supplementary material for: Direct observation of cytoskeleton-dependent trafficking of miRNA visualized by the introduction of pre-miRNA
Source: iScience. 2024 Jan 5;27(2):108811. doi: 10.1016/j.isci.2024.108811 (PMC10831896; doi:10.1016/j.isci.2024.108811)
Supplement: Document S1. Figures S1–S6 and Tables S1 and S2 [file mmc1.pdf]

**iScience, Volume *27***

## Supplemental information

# Direct observation of cytoskeleton-dependent trafficking of miRNA visualized by the introduction of pre-miRNA

**Toshinari Ishikawa, Ko Sugawara, Junwei Zhang, Takashi Funatsu, and Kohki Okabe**

## Supplementary Figures

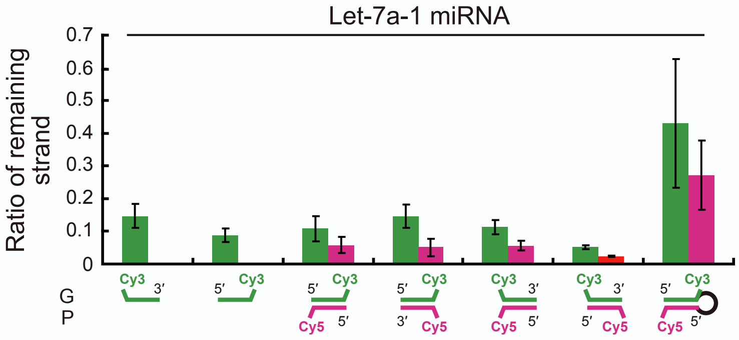


**Supplementary Figure S1. Comparison of remaining efficiency upon introduction of various fluorescently labeled miRNA precursors in living HeLa cells, Related to Figure 1**

The proportion of remaining strands in the cytoplasm. The results are shown for seven miRNA precursors with guide strands labeled with Cy3 and/or passenger strands labeled with Cy5. The proportion of total fluorescence intensity in the cytoplasm at 60 min after microinjection to that at 2 min after microinjection is shown. Guide (green) and passenger (magenta) strands are shown. Error bars represent SD ($n$ = 6–12 cells). G and P stand for guide and passenger sequences of miRNA, respectively.

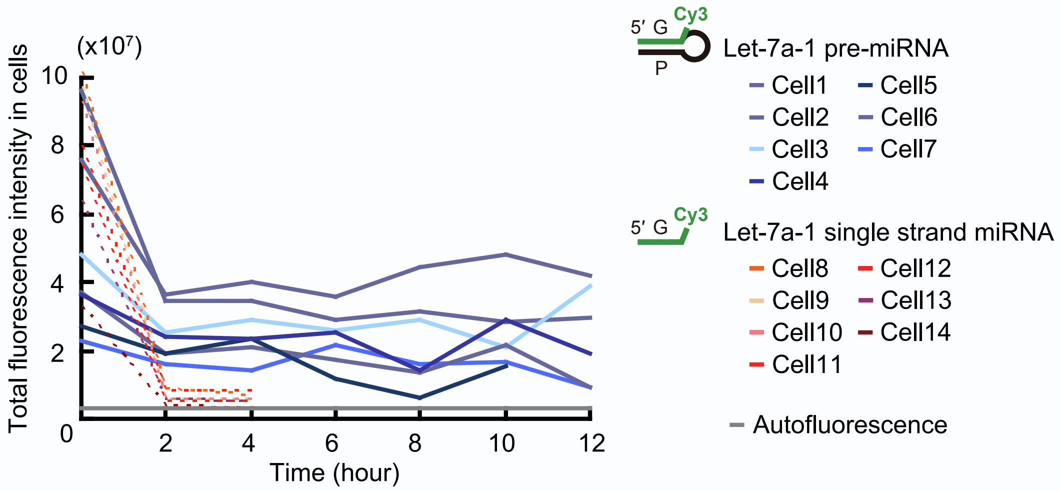


**Supplementary Figure S2. Stability of miRNA precursors in living cells, Related to Figure 1**

The total fluorescence intensity of let-7a-1 miRNA precursors whose guide strand was labeled with Cy3 microinjected into the cytoplasm of living COS7 cells [cells 1–7 (pre-miRNA) and cells 8–14 (single-strand miRNA)] was tracked using epifluorescence microscopy. The signal of autofluorescence is shown as a green solid line. G and P stand for guide and passenger sequences of miRNA, respectively.

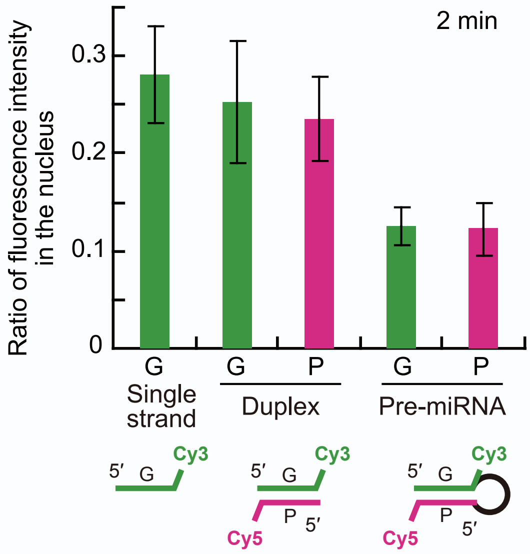


**Supplementary Figure S3. Accumulation of miRNA precursors in the nucleus immediately after introduction, Related to Figure 1**

The proportion of fluorescence intensity of Cy3 (and Cy5)-labeled let-7a-1 miRNA precursors (single-stranded miRNA, duplex, and pre-miRNA) in the nucleus relative to that of the whole cell at 2 min after introduction into the cytoplasm of living COS7 cells. Green and magenta indicate Cy3-labeled guide strand and Cy5-labeled passenger strand, respectively. Error bars represent SD [$n$ = 11 cells (single strand), 12 cells (duplex), 14 cells (pre-miRNA)]. G and P stand for guide and passenger sequences of miRNA, respectively.

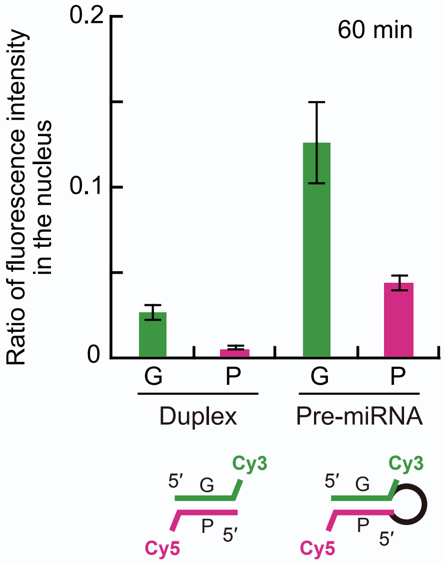


**Supplementary Figure S4. Accumulation of miRNA precursors in the nucleus at 60 min after introduction, Related to Figure 2**

The proportion of fluorescence intensity of Cy3 (and Cy5)-labeled let-7a-1 miRNA precursors (duplex and pre-miRNA) in the nucleus relative to that of the whole cell at 60 min after introduction into the cytoplasm of living COS7 cells. Green and magenta indicate Cy3-labeled guide strand and Cy5-labeled passenger strand, respectively. Error bars represent SD [$n$ = 12 cells (duplex), 14 cells (pre-miRNA)]. G and P stand for guide and passenger sequences of miRNA, respectively.

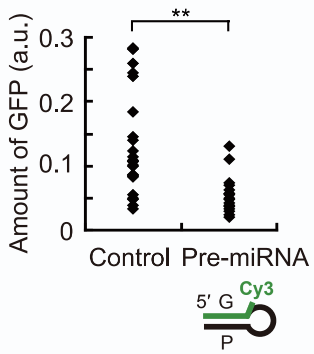


**Supplementary Figure S5. Inhibition of translation by fluorescent miRNA, Related to Figure 2**

The level of GFP of the gene containing the target sequence of let-7a-1 miRNA was normalized to that of RFP that did not have an miRNA target. COS7 cells in which Cy3-labeled let-7a-1 pre-miRNA was introduced showed a significantly lower level of GFP. The control represents the results in cells without pre-miRNA introduction. Each rhombus represents a single cell. $^{**}p < 0.01$; Mann–Whitney U test, $n = 25$ cells (Control) and 17 cells (Pre-miRNA). G and P stand for guide and passenger sequences of miRNA, respectively.

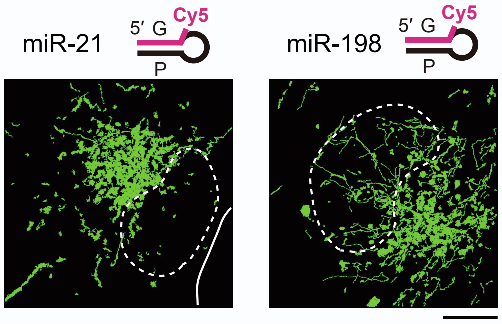


**Supplementary Figure S6. Tracking of miR-21 and miR-198 particles in living cells, Related to Figure**

Cy5-labeled pre-miRNAs were introduced and allowed to mature, followed by single-particle tracking. Dotted and solid lines indicate the nucleus and the cell periphery, respectively. G and P stand for guide and passenger sequences of miRNA, respectively. Scale bar, 10 µm.

# Supplementary Tables

**Supplementary Table S1 Sequences of miRNA used in this study, Related to Figure 1**

| Name | Form | Guide/Passenger | Sequence* |
|---|---|---|---|
| **Let-7a-1** | Single strand | Guide | UGAGGUAGUAGGUUGUAUAGUU-Cy3 |
| | Duplex | Guide | UGAGGUAGUAGGUUGUAUAGUU-Cy3 |
| | | Passenger | CUAUACAAUCUACUGUCUUUC - Cy5 |
| | Pre-miRNA | Guide | UGAGGUAGUAGGUUGUAUAGUU(Alexa488)UUAGGGUCACACC† |
| | | Guide | UGAGGUAGUAGGUUGUAUAGUU(Cy3)UUAGGGUCACACC† |
| | | Guide | UGAGGUAGUAGGUUGUAUAGUU(Cy5)UUAGGGUCACACC† |
| | | Guide | UGAGGUAGUAGGUUGUAUAGUU(Amino)UUAGGGUCACACC† |
| | | Passenger | $PO_3$-CACCACUGGGAGAUAACUAUACAAUCUACUGUCUUU(Cy5)CC† |
| | | Passenger | $PO_3$-CACCACUGGGAGAUAACUAUACAAUCUACUGUCUUUCC |
| **miR-21** | Pre-miRNA | Guide | UGUCGGGUAGCUUAUCAGACUGAUGUU(Amino)GACUGUUGA† |
| | | Passenger | $PO_3$-AUCUCAUGGCAACACCAGUCGAUGGGCUGUCUGACA |
| **miR-198** | Pre-miRNA | Guide | UCAUUGGUCCAGAGGGGAGAUAGGUU(Amino)CCUGUGAU† |
| | | Passenger | $PO_3$-UUUUCCUUCUUCUCUAUAGAAUAAAUGA |

* All sequences are listed from their 5′-end to their 3′-end.

† (Alexa488), (Cy3), and (Cy5) indicate intrachain labelling with corresponding moieties.

**Supplementary Table S2 Sequences of antisense 2′-*O*-methyl RNA oligonucleotides used in this study, Related to Figure 2**

| Target mRNA | Sequence* |
|---|---|
| **Kras (1)** | Amino-AGGCACUCUUGCCUACGCCA-biotin† |
| **Kras (2)** | Amino-UCAUGUACUGGUCCCUCAUU-biotin† |
| **Kras (3)** | Amino-CCUACUAGGACCAUAGGUAC-biotin† |
| **Luciferase‡** | Amino-GUUGUUUUGGAGCACGGAAA-biotin† |

* All sequences are listed from their 5′-end to their 3′-end.
† Streptavidin was attached to antisense probes via biotin to prevent sequestration into the nucleus.
‡ Control probe that does not have a target in mammalian cells.

## Supplementary Movies

**Supplementary Movie 1. Tracking of miRNA particles along actin filaments in living cells.** Movie of Cy5-labeled let-7a-1 miRNA particles (red, 19 times faster than real time) is merged with averaged image of actin filaments (green).

**Supplementary Movie 2. Tracking of miRNA particles along microtubules in living cells.** Movie of Cy5-labeled let-7a-1 miRNA particles (red, 19 times faster than real time) is merged with averaged image of microtubules (green).

**Supplementary Movie 3. Tracking of miRNA particles in cytoskeleton-disrupted cells.** Movie of Cy5-labeled let-7a-1 miRNA particles in living COS7 cells in which both actin filaments and microtubules are destroyed. The movie runs 19 times faster than real time.
